# Psychosocial and Sociocultural Factors Influencing Antenatal Anxiety and Depression in Non-precarious Migrant Women

**DOI:** 10.3389/fpsyg.2018.01200

**Published:** 2018-07-17

**Authors:** Anna Sharapova, Betty Goguikian Ratcliff

**Affiliations:** Faculty of Psychology and Educational Sciences, University of Geneva, Geneva, Switzerland

**Keywords:** depression, anxiety, pregnancy, migrant, acculturation, culture

## Abstract

The aims of this paper are (1) to assess the role of sociodemographic and psychosocial risk factors on antenatal anxiety (AA) and antenatal depression (AD) in first-generation migrant women in Geneva, as compared to a control group of native Swiss women, and (2) to examine the role of acculturation and other sociocultural factors in the development of antenatal distress in migrant women. A sample of 43 migrant and 41 Swiss pregnant women were recruited during the third trimester of pregnancy. AA was assessed by using the State Trait Anxiety Inventory, and AD by using the Edinburgh Postnatal Depression Scale. Acculturation was assessed as a bidimensional process comprising *attachment to the heritage culture* and *adaptation to the local Swiss culture*, using the Vancouver Index of Acculturation. AA in migrant women was mainly predicted by psychosocial factors, namely socioeconomic status, marital support, family presence in Geneva and parity, while AD was predicted by one dimension of acculturation, i.e., attachment to the heritage culture. Our study can inform perinatal health care professionals about some specific risk factors for antenatal distress in migrant women in order to increase systematic screening procedures.

## Introduction

Transition to motherhood constitutes a profound existential crisis in a woman’s life and may lead to increased vulnerability to mental health problems (Heron et al., 2004; Nanzer, 2009). Antenatal depression (AD) is recognized as a major public health issue (Austin, 2004; World Health Organization, 2009), with about 12–20% of pregnant women presenting depressive symptoms (Marcus et al., 2003; Rich-Edwards et al., 2006; Leigh and Milgrom, 2008; Brittain et al., 2015).

Solid evidence has shown that AD can lead to adverse obstetrical and neonatal outcomes (Marcus et al., 2003; Brittain et al., 2015). It may impact fetal development directly (Field et al., 2003) and later affect the quality of the mother-infant relationship, leading to unforeseen difficulties in the child’s cognitive and emotional development (Foss et al., 2004; Goodman et al., 2011). In addition, AD represents a major risk factor for postpartum depression, with about 50% of antenatally depressed women presenting elevated depressive scores in the postpartum period (Heron et al., 2004; Robertson et al., 2004; Austin et al., 2007). This continuity between antenatal and postpartum depression stresses the importance of early detection and treatment of AD in order to reduce psychological morbidity during pregnancy and after birth (Lee et al., 2007; Leigh and Milgrom, 2008; Lefkovics et al., 2014).

Risk factors for AD overlap with those for postpartum depression, namely, past history of psychopathology, poor marital and social support, stressful life events, low self-esteem, and unwanted pregnancy (Robertson et al., 2004; Biaggi et al., 2016). Some specific risk factors have been identified for AD, however, such as unemployment, low socioeconomic status, young age, domestic violence, and physical health problems during pregnancy (Robertson et al., 2004; Rich-Edwards et al., 2006; Leigh and Milgrom, 2008).

Increasing evidence suggests that comorbid antenatal anxiety (AA) may be a significant feature of AD (Heron et al., 2004; Lee et al., 2007; Teixeira et al., 2009). Anxiety is also known to occur as an independent condition during pregnancy but it has been less studied than depression (Matthey et al., 2003; Dunkel Schetter and Tanner, 2012). In fact, studies show that many women suffer from new onset or aggravation of their anxiety symptoms during pregnancy (Ross and McLean, 2006). Different authors evaluated the prevalence of AA as high as 21–50% (Heron et al., 2004; Faisal-Cury and Rossi Menezes, 2007; Lee et al., 2007). This high prevalence might be due to pregnant women’s increased vulnerability to stress (Biaggi et al., 2016). In addition, pregnant women, especially primiparas, report pregnancy-specific anxiety, such as worries about coping with delivery, pain of childbirth, parenting concerns, bodily changes, and a safe outcome for the infant (Öhmann et al., 2003; Faisal-Cury and Rossi Menezes, 2007; Lobel et al., 2008; Koleva et al., 2011). AA has important negative consequences: it not only represents an independent risk factor for the occurrence of postpartum depression (Heron et al., 2004; Austin et al., 2007), but it has also been associated to negative obstetrical and neonatal outcomes, such as small gestational age, low birth weight, slow fetal growth and development (DiPietro et al., 2002; Dole et al., 2003; Orr et al., 2007), and behavior/emotional problems in the child (O’Connor et al., 2002; Foss et al., 2004; Ronald et al., 2011). Therefore, assessment of both anxiety and depression appears to be a clear clinical need during pregnancy, with the objective of early identification and treatment (Matthey et al., 2003; Austin, 2004).

### AA and AD in Migrant Women

Migrant women are at higher risk than native women for developing AD, with the prevalence of the disorder ranging from 25 to 42% (Zelkowitz et al., 2004, 2008; Lara et al., 2009). They also seem to be at higher risk for AA but research on the subject is lacking. This high prevalence might be due to various environmental and contextual stressors associated with migration. The risk for AA and AD increases in migrant women who have been exposed to pre- or post-migratory traumatic events (Foss et al., 2004) and in those who have a precarious or no legal status and who thus face housing or financial difficulties (Bollini et al., 2009; Gagnon et al., 2010; Goguikian Ratcliff et al., 2015b). The lack of local language proficiency has been identified as another risk factor for AA and AD, as it may lead to social isolation and a feeling of discomfort and incompetence (Zelkowitz et al., 2004). Last but not least, it has been shown that the lack of marital and social support, in particular the lack of the mother (Goguikian Ratcliff et al., 2015a), was related to high levels of antenatal distress (Zelkowitz et al., 2004, 2008; Bina, 2008; Bollini et al., 2009).

Most studies on antenatal distress in migrant women focus on disadvantaged populations, such as women from low-income background (Goguikian Ratcliff et al., 2015b). Little is known about the transition to motherhood in non-precarious migrant women. The few existing studies have shown that migrant women in general, even those living in non-precarious socioeconomic conditions and speaking the local language, are at particularly high risk of developing depression and/or anxiety during the perinatal period (Zelkowitz et al., 2004; Ahmed et al., 2008). This suggests that the migration experience is associated with a set of sociocultural factors influencing the psychological well-being of migrant mothers during pregnancy.

#### Sociocultural Transition and Acculturation

In addition to the environmental risk factors associated with the resettlement process, migration involves the move from one political, socioeconomic and cultural system to another (Team et al., 2009; Goguikian Ratcliff et al., 2015a). Culture here is considered as a set of practices executed in a tangible and observable way by a social group, as well as internal patterns and belief systems, each level mutually reinforcing the other. Furthermore, culture is not a static characteristic of an individual who continually adapts to ever-changing environments. Human development is thus embedded in social networks, interpersonal relations, and local environment. Therefore, culture plays an important role in shaping our identity, and constructs our reality via a process of cultural representations within a specific historical and cultural context. Migrant women, who move from one cultural context to another, have to go through the process of acculturation or adjustment to the host culture, in terms of values, behavior, character, and language skills, with subsequent changes in self-identity (Ryder et al., 2000; Berry, 2003).

Acculturation is a process of cultural modification of an individual or a group by adapting to or borrowing traits from another culture. However, acculturation is not a unidimensional process because it does not necessarily result in assimilation and loss of a person’s ethnic identity. It is a dynamic, ongoing process (Berry, 2003; Beck, 2006). Acculturation has long been viewed as a unidimensional process in which migrants move along the continuum toward the acquisition of the host culture, while losing features of their original culture (Cabassa, 2003). However, it has recently been shown that individuals have multiple independent cultural identities. Thus, acculturation is nowadays defined and measured as a bi- or multidimensional process in which individuals may maintain their heritage culture and at the same time participate in the host culture (Berry, 1997; Ryder et al., 2000; Gibson, 2001; Tadmor et al., 2009).

The relationship between acculturation and mental health is complex. Multiple studies have shown that assimilation or acculturation to the host culture is associated with better adjustment in the host country and mental health (see Berry, 1994, for an overview). Furthermore, it has been argued that the biculturalism (successful adaptation to two or more cultures) reflects cognitive flexibility and is thought to be potential resilience factor (Kim and Omizo, 2006; Spiegler and Leyendecker, 2017).

Pregnant migrant women, especially primiparas, are experiencing overlapping life transitions: a sociocultural transition calling for acculturation process and a developmental transition to motherhood (Foss et al., 2004; Goguikian Ratcliff et al., 2015a). During pregnancy, culture determines how the future mother will perceive her pregnancy and prepare for her motherhood. It links the woman to her social group, providing her with contextual cues, resources, and a sense of family continuity (DeSouza, 2014; Goguikian Ratcliff et al., 2015a). Far from their extended families, women cannot turn to older female relatives and friends who can provide a sense of belonging to a family and transmit maternal know-how (Bina, 2008; Baubet and Moro, 2013). This lack of family and cultural resources can become a source of uncertainty, a feeling of maternal incompetence, and emotional distress (Berry, 1997; Koneru et al., 2007; Baubet and Moro, 2013).

The role of acculturation process in the development of AA and AD has so far received scarce attention (Beck, 2006; Fung and Dennis, 2010). The existing studies mainly focused on *postpartum* depression in *second-generation* migrant women (Martinez-Schallmoser et al., 2003). They showed that women born in the host country and highly acculturated to the local culture reported higher postpartum depressive symptomatology, as they were more involved in health risk behaviors (smoking, bad eating habits) and had poorer perinatal outcomes than their less acculturated counterparts. The few studies in first-generation women showed inconsistent results. Some of them reported that the lack of acculturation to the host culture was not related to postpartum depression (see Beck, 2006, for a review), while others concluded that higher levels of acculturation to the host culture was associated with fewer depressive symptoms after birth (Zelkowitz and Millet, 1995; Foss, 2001). The heterogeneity of experimental designs and the variables used to define acculturation limit the conclusions that can be drawn.

In perinatal research, most studies on first-generation migrant women adopt the unidimensional model of acculturation and use various unidimensional scales or proxy measures of assessment. For example, Zelkowitz and Millet (1995) used language proficiency as a proxy measure of acculturation to the Canadian culture. They concluded that the lack of local language proficiency resulted in social isolation and thus led to postpartum depressive symptoms in migrant women. However, studies using the unidimensional measures of acculturation do not inform about the role of maintaining one’s heritage culture in the development of depressive symptoms during pregnancy and after birth. One study (Abbott and Williams, 2006) measured acculturation as a bidimensional process and showed that migrant women in New Zealand were at risk of postpartum depression if they rejected the values of both their heritage and host cultures. Women who maintained their heritage culture and at the same time participated in the host culture were less likely to suffer from depression. Therefore, cognitive flexibility, openness to the host culture, but also preservation of traditional cultural practices in the migratory context, might represent protective factors against postpartum depression (Kim and Omizo, 2006; Bina, 2008; Moro and Drain, 2009; Goguikian Ratcliff et al., 2015a). More research using the bidimensional model of acculturation is necessary, however, to confirm these results and understand how the adaptation to the host culture and the conservation of the heritage culture act as protective factors against depression and anxiety during the entire perinatal period (Beck, 2006; Fung and Dennis, 2010).

## Aims of the Study

The aim of the study was to disentangle sociodemographic, psychosocial and sociocultural risk factors for AA and AD in migrant women in Geneva. The transition to motherhood and antenatal distress in non-precarious migrant women have received scarce attention so far, as most of the studies have focused on disadvantaged populations (refugees, undocumented, or low-income migrant women). In this study, migrant women were compared to native Swiss women of the similar socioeconomic status in terms of sociodemographic and psychosocial risk factors (age, employment status, social and marital support, presence of family members in Geneva, parity) for AA and AD. Further, in migrant women, the role of acculturation in the development of AA and AD was examined. More specifically, the predictive role of the two dimensions of acculturation, namely, *attachment to the heritage culture* and *adaptation to the Swiss culture*, were independently assessed. The role of other sociocultural risk factors (French proficiency and length of stay in Geneva) on AA and AD was also examined.

## Materials and Methods

### Participants and Procedure

Participants were primipara and multipara mothers recruited through flyers and while attending birth preparation classes at the Maternity Unit of the Geneva University Hospitals and the Arcade des Sages-femmes (a collective of independent midwives). Data were collected between January 2015 and March 2016. Consenting women were contacted by phone to set up an interview date. Migrant women were first-generation migrants, defined as foreign-born women who moved to Switzerland during adulthood (at 18 years old or later). The inclusion criteria were as follows: in the third trimester of pregnancy; aged 18 years and older; English- or French-speaking; married or in a cohabiting relationship. The exclusion criteria were fetal malformation or current diagnosis of a severe psychiatric pathological condition.

Of the 89 women who agreed to participate, two did not meet the inclusion criteria, one woman met the exclusion criteria, and two other women were no longer interested in participating when contacted by phone. The final sample consisted of 84 women, 43 of whom were migrants. The study procedure was approved by the Faculty of Psychology Ethical Committee at the University of Geneva.

### Measures

A 21-item sociodemographic questionnaire was used to obtain sociodemographic data (age, country of birth, education level, length of stay in Geneva, type of residence permit, French language proficiency) and psychosocial living conditions (housing, work rate, presence of family members in Geneva). Educational levels of women and their partners were added together (1 = primary education, 2 = secondary education, and 3 = higher education), as well as their work rates (1 = not working, 2 = part-time job, and 3 = full-time job), in order to obtain an indirect measure of their *socioeconomic status*.

The *Edinburgh Postnatal Depression Scale* (EPDS; Cox et al., 1987) was used to screen for AD. It is a 10-item self-report psychometric measure for antenatal and postpartum depression, an instrument of choice in international perinatal research (Cox and Holden, 1994). There is some controversy as to the appropriate cutoff point to use during pregnancy. Murray and Cox (1990) recommended using a cutoff point of 12/13 to screen for both major and minor depression. In this study, we chose to use a cutoff of 10/11, as recommended by the American Pediatric Association, in order to identify women *at risk* for AD. The French version used in this study was validated in France by Guedeney and Fermanian (1998) for the detection of postpartum depression. In the current sample, the Cronbach’s alpha of the scale was 0.77.

The *State-Trait Anxiety Inventory* (STAI; Spielberger, 1983) was used as it allows to assess two distinct anxiety concepts: anxiety as an emotional state (*state anxiety subscale*) and proneness to anxiety as a personality trait (*trait anxiety subscale*), with 20 items on both subscales. Items were scored on a four-point scale (from 1 to 4) with general scores varying from 20 to 80. It is acknowledged that anxiety is low up to a score of 45, moderate between 46 and 55, and high when the score is 56 or more. In French-speaking women, a French version of the STAI validated by Bruchon-Schweitzer and Paulhan (1993) was used. In the current sample, the scale showed good internal consistency, with Cronbach’s alphas of 0.91 and 0.89 for *state anxiety subscale* and *trait anxiety subscale*, respectively.

The *Short-Form Social Support Questionnaire* (SSQ-6; Sarason et al., 1987) was used to measure perceived social support. Two scores were obtained: the extent of social support, i.e., the number of people available in case of need, and the degree of satisfaction with the available support scored on a four-point scale with scores ranging from 6 to 36 points. The French version used was translated and validated by Bruchon-Schweitzer and Paulhan (1993). Coefficient alphas for the current sample were 0.91 for the subscale of the extent of social support and 0.89 for the subscale of satisfaction with the available support.

*Marital support* was measured with two questions: (1) Does your husband/partner provide you with practical and emotional support during pregnancy? (2) How satisfied are you with your husband’s/partner’s support during pregnancy? The second question was rated on a 10-point Likert scale ranging from 0 (not at all satisfied) to 10 (extremely satisfied). The score was used in its continuous form for the analyses.

*Vancouver Index of Acculturation* (VIA; Ryder et al., 2000) was used to access acculturation. It is a 20-item self-report scale that comprises two orthogonal dimensions of acculturation: the extent of attachment to individual’s heritage culture and the extent of adaptation to Swiss mainstream culture. Items have response formats of 1 (disagree) to 9 (agree), addressing several domains of cultural identification, such as social and leisure activities, humor, and adherence to cultural traditions. Scores range from 10 to 90 on each subscale, with higher scores indicating greater identification with each domain. The items of the VIA were adapted to the present sample by replacing the term “American” by “Swiss.” The French version used in this study was created in Belgium by Friedman and Saroglou (2010). In this study, Cronbach’s alpha coefficients were 0.76 for the heritage culture subscale and 0.82 for the Swiss culture subscale.

### Data Analysis

Mean-level differences between migrant and Swiss women for all scales were tested using ANOVAs in Statistica, release 11. Multiple regression analyses were performed to test if different sociodemographic, psychosocial and sociocultural factors predicted AA and AD in the two groups. For migrant women, the role of acculturation was assessed by introducing into the regression analysis the two independent subscales of the VIA, namely, attachment to the heritage culture and adaptation to the Swiss culture. The other sociocultural factors examined were French proficiency and length of stay in Geneva. The scores of the scales were used in their continuous form for the analyses. Multicollinearity was not found to be present in any of the analyses. All analyses were conducted with a significance threshold of α = 0.05, two-tailed.

AA significantly correlated with AD (*r* = 0.70; *p* < 0.001) in all women. AA (measured by *state anxiety subscale* of the STAI) was controlled for in all analyses where AD represented the outcome variable. Proneness to anxiety (measured by *trait anxiety subscale* of the STAI) was controlled for in all analyses where AA represented the outcome variable.

## Results

### Sample Description

A total of 84 women participated in the study. Descriptive analyses revealed that participants ranged in age from 21 to 42 years (*M* = 32.7, *SD* = 4.3). The majority of women were married (*n* = 63, 75%) and the others were in a cohabiting relationship. For most women, the current pregnancy was their first (*n* = 66, 79%) and for 92% of women (*n* = 77), the pregnancy was voluntary. The level of education of participants was high (12% with a Ph.D. or postgraduate degree, 51% with a master’s degree, 20% with a bachelor’s degree). The majority of women (*n* = 66, 79%) were employed, and most of them (*n* = 53, 63%) worked at least 4 days a week. Of the women’s partners, 94% of them were employed.

Migrant women (*n* = 43) did not significantly differ from native women in terms of age, education level, social support, or employment status (see **Table 1**). However, migrant women were more likely to be married (91 vs. 56%, χ^2^ = 13.42, *p* < 0.01) and expecting their first baby (91 vs. 68%, χ^2^ = 6.84, *p* < 0.05). Not surprisingly, they were less likely to have family members in Geneva (7 vs. 88%, χ^2^ = 55.13, *p* < 0.001). They had been living in Switzerland for an average of 5 years (*SD* = 3.9; minimum 5 months and maximum 15 years). Eleven women (26%) had been living in Geneva for less than 2 years, 17 (40%) between 2 and 5 years, and 15 (35%) for 5 years or more. Nearly half of the migrant women (*n* = 18, 42%) came from Western European countries, 10 (23%) came from other OECD countries, such as Canada, Japan, United States; and 15 (35%) came from non-OECD countries, such as Russia, Ukraine, India, Taiwan, and Kenya. Nearly half of the migrant women spoke fluent French (47%), 12% reported limited French proficiency, and 41% had little or no French proficiency. Most migrant women (71%) had a long-term residence permit; 2% had received Swiss nationality; 25% possessed a special permit related to their workplace, such as international and intergovernmental organizations; and 2% had a short-term work permit of less than 1 year.

**Table 1:** Comparison of demographic information for native and migrant participants.

Demographic variable	Migrant (*N* = 43)	Native (*N* = 41)
Mean age of participants (SD)	33.5 (4.2)	31.7 (4.4)
Primiparity (%)^∗^	88.6	67.5
Education (%)		
PhD or postgraduate	14	14
Master’s degree	58	62
Bachelor’s degree	26	21
High school diploma	2	3
Civil status: married (%)^∗∗^	91	53
Percentage of women employed	73	88
Have friends in Geneva (%)	88.6	95
Have family in Geneva (%)^∗∗∗^	6.8	88
Mean EPDS score (SD)	5.8 (3.6)	5.9 (3.7)
Mean STAI state anxiety score (SD)	36.3 (8.8)	36.6 (8.6)
Mean STAI trait anxiety score (SD)	35 (6.8)	35.8 (9.6)
Mean SSQ score		
Number of people available (SD)	28.6 (14)	32.6 (11.8)
Satisfaction with social support (SD)	31.9 (4.2)	33.2 (2.6)
Mean satisfaction with marital support (SD)	8.4 (1.2)	8.8 (1.3)

*EPDS, Edinburgh Prenatal Depression Scale; *STAI*, State-Trait Anxiety Inventory *SSQ*, Short-Form Social Support Questionnaire. ^∗^*p* < 0.05, ^∗∗^*p* < 0.01, ^∗∗∗^*p* < 0.001*.

### Mean Scores

For the current sample, the EPDS and the STAI scores were normally distributed (Skewness < 1, Kurtosis < 1). The mean EPDS score was 5.8 (*SD* = 3.7). Fifteen women (18%) presented a score equal to or more than 10 and were considered at risk for AD. The mean score of the STAI state anxiety was 36 (*SD* = 8.7), and the mean score of the STAI trait anxiety was 34.9 (*SD* = 6.8). Sixteen women (19%) scored 46 or above and thus were experiencing a moderate to high level of anxiety during pregnancy. There was no significant difference in the mean scores of the EPDS or the STAI between migrant and native women (see **Table 1**).

The majority of migrant women were highly attached to their heritage culture, with the mean score of 72.64 (*SD* = 10.7) on the corresponding VIA subscale. They also demonstrated relatively high adaptation to the Swiss culture, with the mean score of 62 (*SD* = 13.2). The attachment to the heritage culture decreased with time spent in Geneva (*b* = 0.32, *p* < 0.05). Unexpectedly, the adaptation to the Swiss culture did not increase over time.

### Sociodemographic and Psychosocial Predictors of AA and AD

*In migrant women*, AA was significantly predicted by a set of sociodemographic and psychosocial factors [*F*(4,38) = 11.18, *p* < 0.001] provided in **Table 2**. The significant predictor variables were trait anxiety (*b* = 0.59, *p* < 0.001), primiparity (*b* = -0.33, *p* < 0.05), satisfaction with marital support (*b* = -0.29, *p* = 0.030), and socioeconomic status of the couple (*b* = 0.30, *p* < 0.05). These four factors predicted 49% of the AA variance. AD was significantly predicted only by state anxiety (*b* = 0.68, *p* < 0.001), predicting 39% of its variance [*F*(1,42) = 28.75, *p* < 0.001]. Age, education level and social support were not found to be predictive of AA and AD. However, migrant women who had family members in Geneva reported less anxiety symptoms (*b* = -0.33, *p* < 0.05), independently of the available social support.

**Table 2:** Results of multiple regression analysis for sociodemographic and psychosocial predictors of AA in migrant women.

Predictor variables	*b*^∗a^	*SE*^b^	*t*(38)	*p*
Primiparity	-0.33	0.13	-2.59	0.01^∗^
Trait anxiety	0.59	0.11	5.39	0.00^∗∗∗^
Satisfaction with marital support	-0.29	0.13	-2.24	0.03^∗^
Socioeconomic status	0.30	0.13	2.36	0.02^∗^

*AA, antenatal anxiety. ^*a*^Standardized regression coefficients. ^*b*^Standard errors of *b^∗^*. *R* = 0.74, *R*^*2*^ = 0.54, adjusted *R*^*2*^ = 0.49, standard error = 0.17. ^∗^*p* < 0.05, ^∗∗∗^*p* < 0.001*.

*In native women*, AA was significantly predicted only by trait anxiety (*b* = 0.73, *p* < 0.001), even when controlling for other sociodemographic and psychosocial factors. AD was significantly predicted by state anxiety (*b* = 0.78, *p* < 0.001) and socioeconomic status (*b* = -0.22, *p* < 0.05), predicting 69% of the variation of AD [*F*(2,38) = 48.54, *p* < 0.001]. In other words, women who presented a lower socioeconomic status and those who were feeling anxious during pregnancy also presented more depressive symptoms.

### Sociocultural Predictors of AA and AD in Migrant Women

When the two independent subscales of the VIA were added to the regression analysis, AA in migrant women was still significantly predicted by trait anxiety (*b* = 0.57, *p* < 0.001) and *psychosocial* factors, namely satisfaction with marital support (*b* = -0.39, *p* < 0.05), and by socioeconomic status (*b* = -0.27, *p* < 0.05). Adaptation to the Swiss culture and attachment to the heritage culture were not found to be predictive of AA, nor was length of stay in Geneva.

As for AD, once the two VIA subscales were added to the regression analysis, it was still significantly predicted by state anxiety (*b* = 0.60, *p* < 0.001), but also by attachment to the heritage culture (*b* = 0.23*, p* < 0.05), independently of the adaptation to the Swiss culture (see **Table 3**). That is, independently of being an anxious person, migrant women presented more depressive symptoms when they were attached to their heritage culture. Both state anxiety and attachment to the heritage culture altogether explained 49% of AD variance [*F*(4,39) = 11.52, *p* < 0.001]. Attachment to the heritage culture predicted 10% of AD variance. Adaptation to the Swiss culture did not predict AD in migrant women.

**Table 3:** Results of multiple regression analysis for psychosocial and sociocultural predictors of AD in migrant women.

Predictor variables	*b*^∗a^	*SE*^b^	*t*(37)	*p*
State anxiety	0.69	0.29	6.31	0.00^∗∗∗^
Socioeconomic status	-0.19	0.11	-1.69	0.10
Attachment to heritage culture	0.21	0.11	1.87	0.04^∗^
Adaptation to Swiss culture	-0.11	0.11	-1.00	0.34

*AD, antenatal depression. ^*a*^Standardized regression coefficients. ^*b*^Standard errors of *b^∗^*. *R* = 0.75, *R*^*2*^ = 0.57, adjusted *R*^*2*^ = 0.52, standard error = 2.55. ^∗^*p* < 0.05, ^∗∗∗^*p* < 0.001*.

The length of stay in Geneva tended to predict AD, but did not reach statistical significance (*b* = -0.21, *p* = 0.069), suggesting that women living in Geneva for a longer period of time tended to present less depressive symptoms, which may be due to the decrease in the attachment to the heritage over time (*b* = 0.32, *p* < 0.05).

French proficiency did not predict AA or AD in migrant women.

## Discussion

In this study, first-generation, non-precarious migrant women were first compared to a control group of Swiss women of the similar socioeconomic status in terms of sociodemographic and psychosocial risk factors for AA and AD, such as age, parity, employment status, social and marital support. Second, the role of acculturation in the development of AA and AD in migrant women was assessed. Acculturation was considered as a bidimensional process that included *attachment to the heritage culture* and *adaptation to the Swiss culture*. The influence of other sociocultural factors, such as French proficiency and length of stay in Geneva, on the development of AA and AD was also examined. The novel feature of this study is the disentanglement of sociodemographic and psychosocial risk factors for AA and AD in migrant women on one hand, and sociocultural risk factors on the other hand.

Migrant women in our sample did not differ from native women in terms of age, education, employment status, socioeconomic environment, or availability of social support. The majority of them came from Western European countries and had a long-term residence permit. The present sample seems to be representative of the general migrant population in Geneva, with two-thirds of permanent migrants coming from Western Europe Federal Statistical (Office of Statistics of the City of Geneva [OCSTAT], 2013). In this study, migrant women were more likely to be married and expecting their first child. The high marriage rates in this sample might be explained, partially at least, by its practical utility in simplifying legal procedures when migrating as a family. As for the delay in transition to motherhood, it might reflect the challenge of becoming a mother while dedicating themselves to their careers in a foreign land (Nunes-Reichel and Santiago-Delefosse, 2015). Not surprisingly in this context, almost all pregnancies were voluntary.

### AA and AD

The rates of AA and AD symptoms in the current sample of migrant women were lower than in those reported in other studies (18 vs. 25–42%) (Zelkowitz et al., 2004; Zelkowitz, 2007; Ahmed et al., 2008). Native women presented similar AA and AD rates as reported in other studies on general population (Faisal-Cury and Rossi Menezes, 2007; Lee et al., 2007; Lara et al., 2009). Contrary to our expectations, migrant women did not differ from Swiss women in terms of rates of depression symptoms and anxiety symptoms. In fact, numerous studies in North American and European literature indicate a higher prevalence of AA and AD among migrants (Foss et al., 2004; Zelkowitz et al., 2008; Lara et al., 2009; Goguikian Ratcliff et al., 2015a). This difference has been explained by the fact that migrant women usually face multiple stressors such as low socioeconomic position, poor social support, and/or difficulties in adaptation to the host culture (Gagnon et al., 2010). In our sample, women did not face any of these factors. Most of them were employed and had high socioeconomic status. Women also benefitted from a solid social network, which constitutes a protective factor against antenatal distress (Martinez-Schallmoser et al., 2003; Zelkowitz et al., 2008; Goguikian Ratcliff et al., 2015b). This result is surprising, as the majority of similar studies show that migrant women often lack social support and report a feeling of isolation (Zelkowitz et al., 2004; Bina, 2008; Goguikian Ratcliff et al., 2015b). The strong social network of migrant women in our sample could be explained by their length of stay in Geneva (the majority of women had been living in Geneva for more than 2 years), their employment status, and the international environment where they lived. In fact, Geneva hosts more than 20 international and intergovernmental organizations, and one third of the active population in the city speak English at work (Office of Statistics of the City of Geneva [OCSTAT], 2013). Thus, even women who did not speak French could create a solid network, often consisting of colleagues or a foreign community. As for acculturation to the local culture, it seems that migrant women in our sample who lived in such an international environment did not feel the need to engage in profound adaptation of the Swiss traditions and values. This result will be further discussed in the following section.

Antenatal anxiety was highly comorbid with AD, in consonance with the conclusions of prior studies (Austin, 2004; Heron et al., 2004). It is still unclear if anxiety and depression constitute clearly separate disorders during pregnancy (Matthey et al., 2003; Dunkel Schetter and Tanner, 2012). In this study, although AA and AD were highly correlated, they might be seen as separate phenomena, as they were predicted by different sociodemographic, psychosocial and sociocultural factors. Again it seems important to separately screen for AA and AD, as they have been shown to independently predict postpartum depression and adverse neonatal outcomes (Foss et al., 2004; Heron et al., 2004; Orr et al., 2007).

### Sociodemographic, Psychosocial and Sociocultural Risk Factors in Migrant Women

Compared to native women in whom AD was predicted by lower socioeconomic status, migrant women were at risk for AD when they were strongly attached to their heritage culture, after controlling for their socioeconomic status and AA. The predictive role of the attachment to the heritage culture in the development of AD is an important finding of this study because it contributes to a better understanding of migrant women’s increased vulnerability to antenatal distress. This result is inconsistent with the findings of Abbott and Williams (2006) in New Zealand, who showed that the integrative or bicultural acculturation strategy (attachment to the heritage culture *and* adaptation to the host culture) represented a *protective* factor against postpartum depression. However, it is difficult to compare results of the present study with those of the study by Abbott and Williams (2006), as the attachment to the heritage culture in their study was assessed only with regard to adaptation to the local culture, but not independently. Thus the interdependent nature of the two subscales did not permit to access the particular role of each acculturation dimension separately. Moreover, the authors focused on postpartum depression in Pacific mothers of low socioeconomic status, thus confounding socioeconomic and sociocultural factors.

The result of our study underlines the fact that for the first-generation, non-precarious migrant women in our sample, attachment to their heritage culture represented a source of increased vulnerability, as their transition to motherhood occurred far from their families and cultural framework. In a new cultural and social context, their sense of belonging might have been undermined, especially since they experienced overlapping transitions (migration and pregnancy). Pregnancy is a period when the need to belong to a family system and to continue its heritage is absolutely crucial (Bina, 2008; Baubet and Moro, 2013). Migrant women are far from their mothers and larger family, and from the usual intergenerational transmission and guidance practices, which may lead to a loss of the sense of family anchorage. Migrant mothers have to reconcile the two cultures during the acculturation process, and come up with a new, bicultural identity (Koneru et al., 2007; DeSouza, 2014). In this context, the strong attachment to the heritage culture in migrant women in our sample might reflect their longing for the lost familiar cultural context and family ties. This hypothesis is further supported by the fact that migrant women who had family members living in Geneva reported significantly less anxiety symptoms than women who did not. Women who kept their cultural traditions and beliefs might have also faced some discrepancies between their cultural practices related to pregnancy (breastfeeding, sleeping routines, and child rearing practices) and local guidelines given by health care professionals. In her literature review on the impact of cultural factors on postpartum depression, Bina (2008) concluded that the maintenance of cultural traditions in the host country could be at the same time a risk factor and a protective factor against depressive symptoms; therefore, the role of attachment to the heritage culture varies among individuals and contexts.

As for AA in migrant women, it was mainly predicted by trait anxiety (proneness to anxiety as a personality trait), and sociodemographic and psychosocial variables, namely primiparity, low satisfaction with marital support, and low socioeconomic status. These results confirm the findings of prior studies, stressing that in the context of migration, partner’s support takes on even greater significance (Zelkowitz et al., 2004; Ahmed et al., 2008; Bina, 2008). Low socioeconomic status represents a major risk factor for antenatal distress (Robertson et al., 2004; Bollini et al., 2009). Migrant women in our sample often expressed fears of losing their position after the birth of the baby, and thus encounter financial difficulties. This was particularly true for women whose legal permit in Switzerland was related to their workplace (a quarter of migrant women).

Even though most migrant women in our sample adapted to the Swiss culture, those women who were less integrated or had little French proficiency did not report higher anxiety or depression symptoms. Lack of knowledge about the host culture can be stressful and a few existing studies on the subject have shown that acculturation to the host culture and higher language proficiency in migrants reduced acculturation stress, depressive symptoms and anxiety (Foss, 2001; Foss et al., 2004; Abbott and Williams, 2006). It should be noted that we used the VIA scale, in which adaptation to the Swiss culture was assessed by one’s knowledge of different areas of local culture, such as cuisine, films, music, and national holidays, as well as socializing with Swiss people. Similarly, in their study on the experience of skilled migrant women in Switzerland, Nunes-Reichel and Santiago-Delefosse (2015) showed that expatriates only spoke English at work and socialized mostly with their colleagues, which constituted at the same time a comfort and an important obstacle to the integration of local practices.

## Conclusion

The present study expands our understanding of the specific role of sociodemographic, psychosocial and sociocultural risk factors on antenatal distress in migrant women. More research is needed to better understand the role of the attachment to the heritage culture and the adaptation to the host culture, assessed separately, in the antenatal distress of migrant women.

### Limitations of the Study

Several limitations of the study need to be highlighted. First, the voluntary nature of participation may have induced a selection bias, as women who decided to participate might be those who felt more satisfied with the pregnancy experience. Second, the sample consisted of women who were married or in couple, with high levels of education and high socioeconomic status, which limits the generalization of the results to all migrant women. Third, we used self-report questionnaires to measure anxiety and depressive symptoms. These are convenient and easy-to-use methods, but they are not appropriate for establishing a diagnosis of depression and anxiety, as they are not designed to do so. The STAI was used to measure anxiety symptoms, as it allows to assess anxiety as an emotional state and proneness to anxiety as a personality trait. Pregnancy specific anxiety scale might have better reflected pregnancy related worries. A psychiatric diagnosis based, for example, on DSM-IV criteria would allow a more accurate estimate of the rates of AA and AD, but the establishment of diagnoses was beyond the purpose of this study.

## Ethics Statement

This study was carried out in accordance with the recommendations of the Ethical Committee of the Faculty of Psychology and Educational Sciences of the University of Geneva. The protocol was approved by the Ethical Committee of the Faculty of Psychology and Educational Sciences. All subjects gave written informed consent in accordance with the Declaration of Helsinki.

## Author Contributions

This study has been conducted in the context of AS’s doctoral thesis. AS participation included the project preparation and elaboration, the obtention of the Ethical Committee agreement, recruitment of participants, antenatal interviews and administration of the questionnaires, data entry and statistical analyses, and article writing and editing. BGR elaboration of the study project, supervision of the study procedure, methods, as well as the article writing; participation in the interpretation of the results and discussion writing.

## Conflict of Interest Statement

The authors declare that the research was conducted in the absence of any commercial or financial relationships that could be construed as a potential conflict of interest.
